# Adaptive laboratory evolution of *Cupriavidus necator* to sodium salts as a whole cell engineering strategy for enhanced trehalose production

**DOI:** 10.1186/s12934-026-03167-8

**Published:** 2026-11-07

**Authors:** Lisa van der Sande, Manuel Müller, Habibu Aliyu, Anne-Kristin Kaster, Dirk Holtmann

**Affiliations:** 1 Process Engineering in Life Science, Electrobiotechnology, Karlsruhe Institute of Technology, Karlsruhe, 76131 Germany; 2 Institute for Biological Interfaces 5, Microbial Genetics & Biotechnology, Karlsruhe Institute of Technology, Eggenstein-Leopoldshafen, 76344 Germany

**Keywords:** *Cupriavidus necator*, Adaptive laboratory evolution (ALE), Compatible solutes, Trehalose production, Osmotic stress

## Abstract

Trehalose is an important compatible solute with diverse applications in the pharmaceutical, food, and cosmetic industries. In *Cupriavidus necator*, trehalose biosynthesis is naturally associated with osmotic stress, but typically retained intracellularly as an osmo-protectant. In this study, adaptive laboratory evolution (ALE) was used as a whole cell engineering strategy to adapt *C. necator* H16 to increasing osmotic stress. ALE was performed with independent lineages and gradually increasing concentrations of NaCl and Na₂SO₄, reaching stable growth at up to 500 mM NaCl or 400 mM Na₂SO₄. In selected single colonies of the adapted cultures, the SetA sugar efflux transporter from *Escherichia coli* was expressed to enable trehalose secretion. Moderate concentrations of Na₂SO₄ were identified as most effective osmotic trigger, balancing growth and trehalose production. Under the selected production conditions the best-performing adapted isolate produced 11.5 g/L trehalose from 20 g/L fructose, corresponding to 60.5% of the theoretical maximum yield, compared to 7.4 g/L and 42.1% in the parental strain. Genome sequencing revealed mutations affecting transporters, membrane structure, metabolism and regulators. These mutations are considered contributors to the observed osmoadaptive phenotype, while individual effects on trehalose production were not resolved. Overall, the results demonstrate the potential of ALE as a whole cell engineering strategy for generating *C. necator* isolates with improved extracellular trehalose production under defined conditions.

**Graphical Abstract:**

**Figure:**
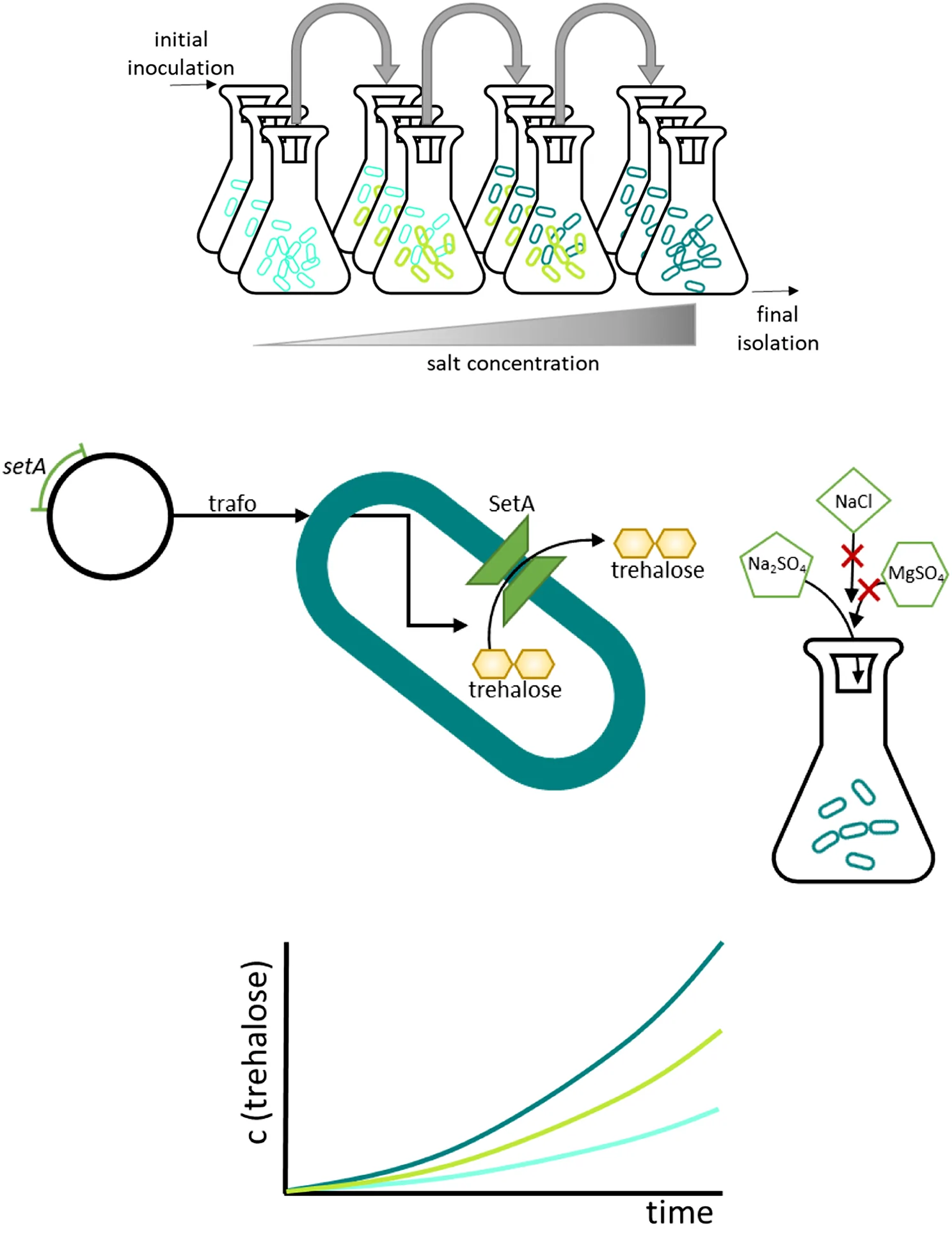

**Supplementary Information:**

The online version contains supplementary material available at https://doi.org/10.1186/s12934-026-03167-8.

## Highlights

*Cupriavidus necator* H16 was adapted to increasing NaCl and Na_2_SO_4_ concentrations by ALE with final concentrations of 500 mM NaCl and 400 mM Na_2_SO_4_.

ALE was used as a whole cell engineering strategy with subsequent selection for osmotic-stress resistance.

In the adapted isolate, the trehalose exporter SetA was expressed using a plasmid.

Moderate Na_2_SO_4_ concentrations of 150 mM provided an effective osmotic trigger for enhanced trehalose production.

The selected NaCl-adapted isolate produced 11.5 g/L trehalose from 20 g/L fructose.

Genome sequencing revealed mutations in genes potentially associated with osmoadaptation.

**Supplementary Information:**

The online version contains supplementary material available at https://doi.org/10.1186/s12934-026-03167-8.

## Background

Microorganisms are confronted with various stressors in their natural environments. To protect themselves against external stress caused by osmotic imbalance, desiccation, temperature and many more, bacteria developed various protective mechanisms. Osmotic stress and extreme desiccation are a huge threat to microorganisms as both cause diffusion of water dehydrating cells until they die. In high osmotic environments the concentration of soluble particles outside a cell tends to be higher than inside it. By laws of diffusion or more specifically osmosis, water flows across the membrane to balance the particle concentration, which causes crucial water loss for the cell. As microorganisms commonly have a high-water content, shown in e.g. *Salmonella enterica*, they developed protective mechanisms against osmotic stress and the following fatal water loss [1].

In general, two strategies to antagonize water loss due to osmotic stress or desiccation developed in single cell organisms. Extremely halophilic archaea and bacteria are known for the “salt in”-strategy, in which ions like K^+^ and Cl^−^ are taken up from the environment to balance the salt gradient between cytoplasm and environment [2]. Cells often import K^+^ and Cl^−^ to keep internal salt concentrations at high, even molar levels, while sodium is kept outside as enhanced sodium concentrations can be toxic for enzymes and disturb the membrane potential [3–5]. This mechanism is relatively energy efficient but requires a special conformation of the intracellular processes [6]. The entire proteome of each cell must be adapted to high ionic strength by becoming highly acidic. With this adaptation such organisms only survive in saline environment [3–5]. In contrast, mesophilic bacteria can’t tolerate high intracellular salt concentrations as the ions interact with protein stability of their proteome, subsequently they developed an alternative mechanism called the “salt-out”-strategy [7]. Therefore, bacteria do not take up salt or their ions into the cytoplasm to lower the water loss, but take up or synthesise so called compatible solutes *de novo* that do not disturb their cellular processes. Compatible solutes, also known as osmo-protectants, are small, neutral, organic molecules like sugars (e.g. trehalose, glycerol, mannitol, sorbitol, sucrose), amino acids (e.g. proline, glutamate, glutamine, ectoine) and quaternary ammonium compounds (e.g. glycine betaine, dimethyl-sulfoniopropionate) that act as osmolytes [8–11]. They help cells survive extreme osmotic stress and remain non-toxic even at high concentrations. When bacterial cells encounter osmotic stress, first it comes to water efflux which reduces turgor pressure [12]. As an initial response similar to halophilic bacteria inmany mesophiles K^+^ ions are taken up from the environment through turgor sensitive potassium-channels. K^+^ ions work as a spark for a cascade like production of compatible solutes [13, 14]. For *Acinetobacter baumannii* König et al. showed that within minutes after an osmotic shock the initial K^+^ uptake started, also the accumulation of glutamate as counterion to potassium, started and peaked shortly afterwards. After glutamate, mannitol and trehalose are formed [14]. Similar mechanisms were also shown in *Escherichia coli* and *Bacillus subtilis* [15–18]. This also shows that trehalose, as it is synthesised *de novo*, is energy intensive in biosynthesis, and therefore is produced not as an initial response but later during continuous osmotic stress. When osmotic stress decreases, those solutes can be secreted, degraded or metabolized again, as especially for trehalose metabolization is a common and energy efficient way [19, 20].

Naturally, trehalose is produced via different pathways. The biosynthesis pathway OtsAB contains two enzymes, the α-trehalose-phosphate synthase (OtsA), which catalyses the formation of trehalose-6-phosphate from UDP-glucose and glucose-6-phosphate and the trehalose-6-phosphate phosphohydrolase (OtsB), which dephosphorylates the intermediate product resulting in trehalose [21]. The TreYZ pathway forms trehalose from glycogen. The TreS pathway forms trehalose from maltose via the trehalose synthase. This single step conversion is reversible and therefore also a common degradation pathway. The TreT pathway is also a single step reversible pathway, producing trehalose via trehalose glycosyl-transferring synthase from glucose and ADP-glucose. A fifth pathway uses the trehalose phosphorylase TreP for trehalose production form glucose and glucose-1-phosphate. TreT and TreP also work as degradation pathways due to their reversibility [22–25].

*Cupriavidus necator* (formerly known as *Ralstonia eutropha* or *Hydrogenomonas eutropha*) is a versatile production host originally found in soil and water [26]. As a ‘knallgas’ bacterium, *C. necator* can undergo heterotrophic, chemolithoautotrophic and mixotrophic metabolism. It has also been used in electro-autotrophic fermentation. With a broad product spectrum it has been extensively investigated for production of value-added compounds from side-streams [27–36]. Trehalose is a common solute under species like *E. coli*,* A. baumannii*, *Corynebacterium glutamicum* or also *C. necator* which all produce it as protective solute under osmotic stress [21, 37, 38]. *C. necator* lacks a natural transporter to secrete trehalose since solute accumulation protects against osmotic stress only while solutes remain intracellular. However, the cosmetic, food and pharma industries are highly interested in solutes like trehalose, making its bacterial secretion advantageous for simplifying downstream processing and achieving higher yields [39, 40].

Trehalose is commonly produced via enzymatic synthesis. In the early 1990s, expenses for production of 1 kg trehalose went up to 700 US $. Currently the production costs via enzymatic synthesis lay around 6 US $ per kilogram. However, due to its numerous applications in the cosmetic, food and pharmaceutical industry e.g. stabilization of antibodies, enzymes, vaccines and even organs during the transplantation process, there was increasing interest in lowering costs for enzymatic production [39–44]. Therefore, research to improve the enzymatic production process was strongly promoted. Enzymatic processes often provide higher efficiency, although the production of enzymes themselves remains costly [45, 46]. As alternative to the enzymatic process to produce trehalose, its production by bacterial fermentation was also investigated, e.g. in a *Micrococcus* wildtype strain [47]. A first step towards making bacterial production more feasible using *C. necator* was the development of strains that secrete trehalose into the medium, eliminating the need for extraction from biomass [37]. As a natural producer *C. necator* was equipped with an efficient transporter here by heterologous overproduction of the sugar efflux transporter A (SetA) of *E.coli*. Trehalose biosynthesis is naturally triggered by desiccation or even more by high osmolarities to counteract water loss. Thus, salts represent both a stress factor and a potential inducer of a valuable product.

Improving a stress-responsive phenotype, such as trehalose production, may involve multiple cellular processes, including compatible solute metabolism, membrane homeostasis, transport, regulation and energy metabolism. Consequently, modification of a single predefined target may not be sufficient to optimize the overall cellular response. Adaptive laboratory evolution (ALE) provides an alternative whole-cell engineering strategy in which cells are subjected to a defined environmental selection pressure over multiple generations. Rather than targeting a predefined gene, ALE enables cells to adapt to imposed conditions by simultaneously modulating multiple cellular functions [48–51]. Previously, ALE was used successfully used to enhance formate consumption and lower the energy demand in bio-electrochemical cultivations in *C. necator* [48, 49]. Also, in other species like *E. coli* multiple successful examples are known like higher tolerance to toxins or also a salt adaptation for improved succinate biosynthesis [50, 52]. For osmotic stress adaptation, ALE is particularly suitable because the cellular response involves multiple interconnected processes. Adaptation to increasing salt concentrations may therefore generate a cellular phenotype that is more efficient at coping with osmotic stress. Such a phenotype could subsequently be exploited for production of stress responsive metabolites without requiring the underlying molecular changes to be known in advance. Following genomic or transcriptomic analysis could enable targeted genetic engineering in the future that could lift production efficiency to whole new levels [53].

Our approach in the present study is, to investigate, whether enhancing the natural salt-stress resistance of *C. necator* via ALE could be used as a whole-cell engineering strategy to generate *C. necator* isolates with improved extracellular trehalose production. ALE was performed in the absence of the heterologous sugar exporter SetA, which was plasmid based introduced afterwards. This separation was intended to select primarily for adaptation to osmotic stress of the whole cell selected by growth at high osmolarities rather than trehalose production as an indirect result of it. After ALE individual adapted colonies were equipped with SetA to enable trehalose export.

The aim of this study was therefore to establish a workflow in which ALE to high salt concentrations is used to generate wholistically engineered *C. necator* variants followed by introduction of SetA and optimization of the osmotic trigger for extracellular trehalose production. After Löwe et al. (2021) introduced the possibility to excrete trehalose, we show a simple way to enhance the yield of trehalose by exploit of the natural production mechanism.

## Experimental procedures

### Culture conditions and media

For heterotrophic growth of all strains (*C. necator* H16 DSM 428 and adapted variants) were incubated at 30 °C with shaking at 180 rpm, 300 mg/L kana mycin was added for transformed strains.

For trehalose production in minimal medium all strains were cultivated in a seed train of two precultures. From a cryo-conserved glycerine-stock stored at -80 °C cells were spread on LB agar plates (5 g/L NaCl, 5 g/L yeast extract, 10 g/L tryptone, 1.8% agar, pH 7) and incubated at 30 °C for 24 h. 5 mL LB-medium (as above, without agar) [54] were inoculated from a single colony and incubated over night at 30 °C and 180 rpm. This preculture was followed by precultures in minimal medium. The minimal medium was composed as described before heterotrophically by the addition of 4 g/L fructose for screening experiments and 20 g/L for final production experiments [55]. The cultures were incubated at 30 °C and 180 rpm for 16 h. Main production-cultures were inoculated at an optical density at a wavelength of 600 nm (OD_600_) of 2. At the time of inoculation 0.1 mM 3-methylbenzoate (3-MB) was added for the expression of *setA *via XylS/Pm, a positively regulated transcription factor activated by benzoate-derived inducers [56], in the plasmid pSEVA228-setA (kindly provided by the Kremling group, TU Munich) to secrete trehalose. Salt was added at the time of inoculation as osmotic trigger for trehalose production by *C. necator*. Na_2_SO_4_, NaCl and MgSO_4_ were initially compared at 150 mM. Based on these screenings Na_2_SO_4_ was selected for further optimization. Na_2_SO_4_ concentration between 0 and 400 mM were tested. Screening experiments shown in figure S2 and S3 were conducted in 96 well plates. The adaptation (Fig. 2 and S1) as well as pre-experiments shown in Fig. 4, S4 and S5 were conducted in small scale shake flasks of 100 mL and 20 mL working volume, the main experiment shown in Fig. 6 in bigger shake flasks of 500 mL and 100 mL working volume.

The salt concentrations used for production were independent from the maximum concentrations reached during ALE. During ALE, high salt concentrations were used as selection pressure, whereas during production moderate salt concentrations were preferred to balance osmotic stress triggering trehalose production and cellular growth.

### Growth analytics

Growth was measured photometrically via OD_600_ in a tabletop photometer (Genesys 30, Thermo Fisher Scientific Inc., USA), as well as the Stratus microplate reader (Cerillo, Charlottesville, VA, USA). Kinetic measurement of growth in the microplate reader was performed at 30 °C at 500 rpm on a Thermomixer C (Eppendorf, Germany).

### Adaptive laboratory evolution (ALE)

**Fig. 1:**
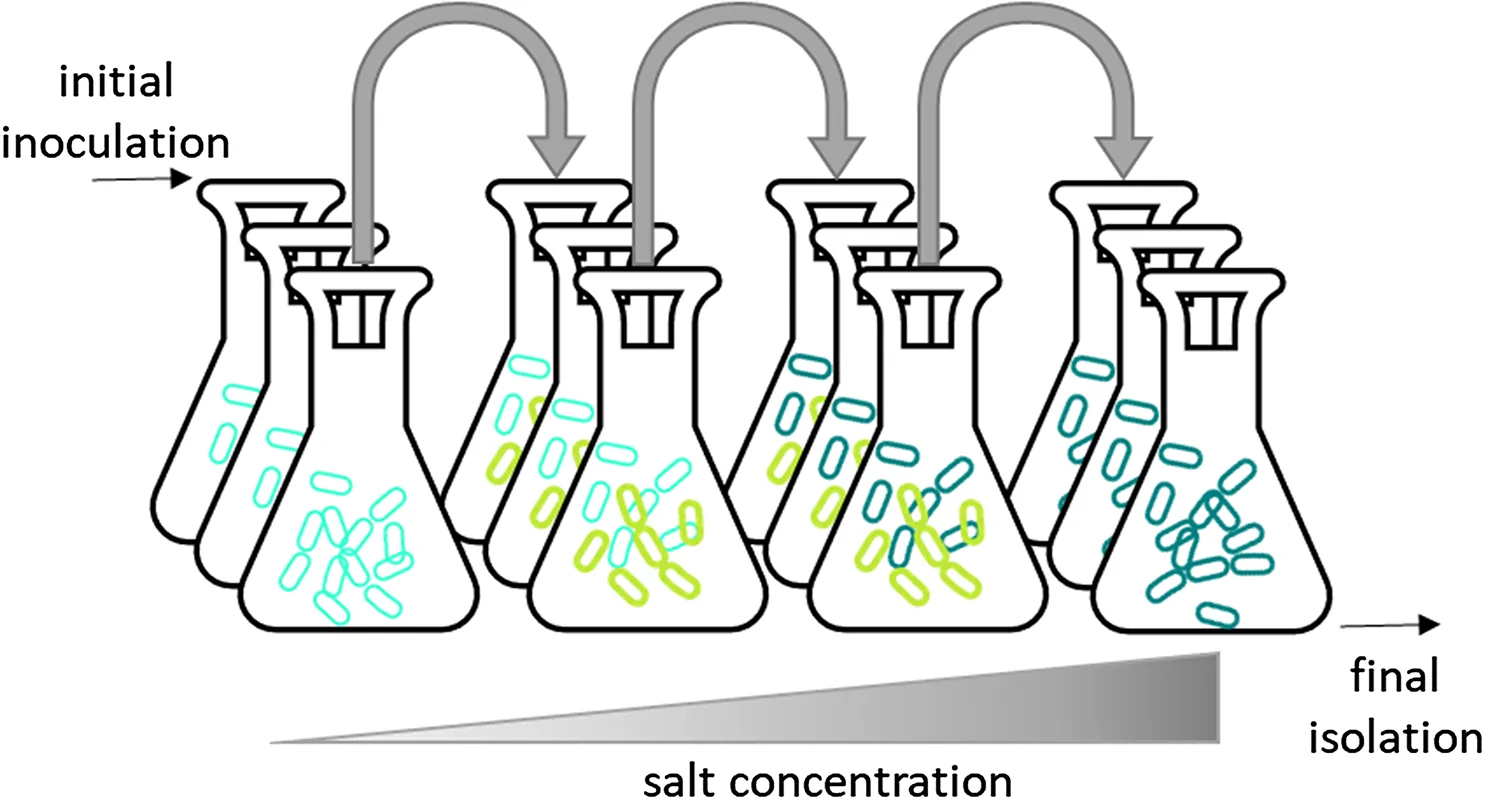
Scheme - Adaptive laboratory evolution

ALE was conducted in 100 mL baffled Erlenmeyer flasks. Cells were grown in heterotrophic minimal medium [55] with 4 g/L fructose as carbon source. Over the duration of 3 months the concentration of Na_2_SO_4_ was increased from 0 to 400 mM or the NaCl-concentration was increased up to 500 mM. The initial steps of concentration enhancement were bigger (100 mM) to speed up the long ALE process, as *C. necator* is naturally able to maintain stable growth at salt concentrations of up to 200 mM. After this concentration was reached, smaller steps (50 mM) were taken, to enable a gentle adaptational process. Cells were transferred to fresh medium daily (Fig. 1). Therefore, the OD_600_ of the culture was measured and 20 mL of fresh medium were inoculated to an OD_600_ of 0.1 [57]. Characterisation of adapted strains was conducted in varying concentrations of Na_2_SO_4_ and NaCl from 0 to 500 mM. For each condition, two independent ALE lineages were initiated and kept separately, one functioning as a backup. Following successful adaptation, cultures were plated on agar plates for separation of individual colonies. The adapted individual colonies were transformed with the plasmid pSEVA-228 enabling trehalose secretion.

### Molecular biology & bioinformatics

#### Plasmid and gDNA isolation

Plasmid DNA was isolated with the Monarch Spin Plasmid Miniprep Kit according to the manufacturer’s instruction (Monarch Spin Plasmid Miniprep Kit, New England Biolabs, NEB Inc., Ipswich, USA). The concentration of plasmid-DNA was measured via NanoDrop (NanoDrop One UV-Vis Spectrophotometer, VWR, Radnor, PA, USA). For sequencing of the adapted and parental strains their DNA was extracted with the Monarch genomic DNA purification kit (Monarch Genomic DNA Purification Kit, New England Biolabs, NEB Inc., Ipswich, USA) according to the manufacturer’s manual. The concentration of the purified DNA was measured using the Qubit dsDNA HS Assay Kit with the Qubit 3.0 Fluorometer (Thermo Fisher Scientific Inc., Waltham, MA, USA).

#### Sequencing and mutation identification

Purified genomic DNA from isolated adapted variants that were used in all experiments, was used to prepare sequencing libraries with the NEBNext Ultra II FS DNA Library Prep Kit for Illumina (New England Biolabs GmbH, Frankfurt am Main, Germany), using unique dual-index adapters, according to the manufacturer’s protocol. Beginning with 100 ng of DNA, the adapter-ligated libraries were PCR-amplified for only 4 cycles to minimize PCR artefacts. Library quality was assessed using the 5200 Fragment Analyser with the DNF-474 HS NGS Fragment Kit (Agilent Technologies GmbH, Waldbronn, Germany). Sequencing was performed in-house on an Illumina NextSeq 1000 (Illumina, San Diego, CA, USA) platform with 2 × 150 bp paired-end chemistry, yielding approximately 100-fold coverage across all samples. Raw read quality was assessed using FastQC v0.12.1 (http://www.bioinformatics.babraham.ac.uk/projects/fastqc/) and further processed with fastp v0.23.4 [58] for adapter removal and quality filtering.

To ensure accurate variant calling, the *C. necator* H16 reference genome (assembly: GCF_004798725.1) was compared to the sequenced genome of the ancestral *C. necator* H16 strain from this study. Mutations present in the ancestral strain were incorporated into the reference using the gdtools in Breseq v0.39.0 [59, 60], resulting in a refined reference genome. Trimmed reads from the adapted strains were then aligned to this refined reference using Breseq in consensus mode with its Bowtie2-based aligner. Single-nucleotide variants (SNVs) and insertions/deletions (indels) were identified using default Breseq parameters. Breseq-identified mutations were subsequently annotated and tabulated using gdtools [59, 60].

#### Strain modification

To transform *C. necator* cells via electroporation they were made electrocompetent (adapted from Lenz et al., 2018) [61]. Small changes from the original protocol were made, including cultivation at 30 °C instead of 37 °C, LB instead of LSLB-medium and measurement of growth at OD_600_ instead of OD_436_.

All used strains, including adapted and transformed strains, are listed in Table 1.

**Table 1:** List of strains used in this study

Strain	Description	Source
*Cupriavidus necator* H16	Type strain DSMZ 428	Schlegel 1961
*Cupriavidus necator* H16 adNaCl	DSMZ 428 adapted to NaCl	this study
*Cupriavidus necator* H16 adNa_2_SO_4_	DSMZ 428 adapted to Na_2_SO_4_	this study
*Cupriavidus necator* H16 SetA	DSMZ 428 with plasmid pSEVA228-setA	Löwe et al. 2021
*Cupriavidus necator* H16 adNaCl SetA	DSMZ 428 adapted to NaCl with plasmid pSEVA228-setA	this study
*Cupriavidus necator* H16 adNa_2_SO_4_ SetA	DSMZ 428 adapted to Na_2_SO_4_ with plasmid pSEVA228-setA	this study

To verify the successful transformation of all strains (*C. necator* H16, H16 adNaCl and H16 adNa_2_SO_4_) with the plasmid pSEVA228-setA a colony-PCR was done with a forward (Seq_setA_fw, sequence (5’ – 3’): CAATAATAATGGAGTCATGACCATGC) and a reverse primer (Seq_setA_rv, sequence (5’ – 3’): AGGGTTTTCCCAGTCACGAC) annealing up and downstream of *setA* on the plasmid, untransformed cells were used as negative control. For the PCR the One*Taq*^®^ Hot Start 2x Master Mix with GC Buffer (NEB-M0485) was used. For a reaction volume of 25 µL 12.5 µL of polymerase master mix and 0.5 µL of each primer (10 µM) were added as well as 2 µL culture of an OD_600_ 3–5 as DNA template, milliQ water was added to a volume of 25 µL. The PCR program was performed according to manufacturer instructions for Taq-polymerase (NEB), with an annealing temperature of 50 °C and an extension time of 1.5 min.

To analyse PCR products of the colony-PCR gel electrophoresis was performed. A 1% agarose gel was used with 30 mL 1x TAE buffer (0.4 M Tris, 0.2 M acidic acid, 0.01 M EDTA, pH 8) and agarose which was dissolved in heat. After cooling down 2 µL of roti gel stain was added before hardening the gel. 10 µL of PCR product was used for gel electrophoresis. As standard 4 µL of the 1 kb DNA-ladder and 100 bp DNA-ladder (NEB) were used. The samples were separated at 150 V for 25 min and afterwards the gel was analysed with the B/G LED-transilluminator. A PCR product of the size if 1350 bps was expected.

### Sugar analytics

Samples were prepared by centrifuging at 14.000 rpm for 10 min and filtering the supernatant with a syringe filter. 100 µL of the samples were transferred into a glass vial with an insert and kept at 4 °C till analysed. Sugars, mainly extracellular trehalose and fructose, were measured, adapted from [62] via HPLC (Agilent 1100 Series, Agilent Technologies, Santa Clara, CA, USA). The mobile phase was 5 mM H_2_SO_4_ at a flow of 0.5 mL/min. The stationary phase was a Rezex ROA-Organic Acid H+ (8%), LC Column, 300 × 7.8 mm (Phenomenex, Aschaffenburg, Germany), with a Rezex ROA-Organic Acid H+ (8%), LC Column, 50 × 7.8 mm used as a precolumn (Phenomenex, Aschaffenburg, Germany), they were heated to 55 °C. For the detection of sugars, a refractive index detector (Agilent G7162A 1260 Infinity II RID, Agilent Technologies, Santa Clara, CA, USA) was used and run at 55 °C. 10 µL samples were run for 20 min, the expected retention time of fructose was 14.7 min and of trehalose 11.45 min. Evaluation and analysis were performed with OpenLAB CDS software of Agilent (Agilent Technologies, Santa Clara, CA, USA).

### Calculations

To calculate the cell dry weight (CDW) from the OD_600_ that was measured, a correlation according to Grunwald, 2015 [63] was used.

To calculate the yield of biomass per substrate (Y_X/S_) Eq. 1 was used:

1 $$\:{\mathrm{Y}}_{\mathrm{X}/\mathrm{S}}=\frac{{\Delta\:}\mathrm{m}\:\mathrm{X}}{{\Delta\:}\mathrm{m}\:\mathrm{S}}\mathrm{*}100{\%}$$

To calculate the yield of product per substrate (Y_P/S_) Eq. 2 was used:

2 $$\:{\mathrm{Y}}_{\mathrm{P}/\mathrm{S}}=\frac{{\Delta\:}\mathrm{m}\:\mathrm{P}}{{\Delta\:}\mathrm{m}\:\mathrm{S}}\mathrm{*}100{\%}$$

To calculate the yield of product per biomass (Y_P/X_) Eq. 3 was used:

3 $$\:{\mathrm{Y}}_{\mathrm{P}/\mathrm{X}}=\frac{{\Delta\:}\mathrm{m}\:\mathrm{P}}{{\Delta\:}\mathrm{m}\:\mathrm{X}}\mathrm{*}100{\%}$$

To calculate the volumetric productivity q_P_ Eq.4 was used, dividing the product concentration through the production duration:

4 $$\:{\mathrm{q}}_{\mathrm{P}}=\frac{{\Delta\:}\mathrm{c}\:\mathrm{P}}{{\Delta\:}\mathrm{t}}$$

To calculate the theoretical maximum trehalose yield from fructose, it was assumed that two moles of fructose with a molar weight of 180.16 g/mol are converted into one mole of trehalose with a molar weight of 342.3 g/mol, without accounting for biomass formation, maintenance or other metabolic losses. 20 g/L or 111 mM of fructose was used which results theoretically in a maximum yield of 18.83 g/L or 55.5 mM of trehalose. For all calculations that were performed Eq. 5 was used:

5 $$\:\mathrm{n}=\frac{\mathrm{m}}{\mathrm{M}}\mathrm{*}\mathrm{V}$$

In a last step, the percentage of the measured trehalose from the theoretical max. yield was calculated using weight or molarity.

## Results & discussion

### Strain modification

**Fig. 2:**
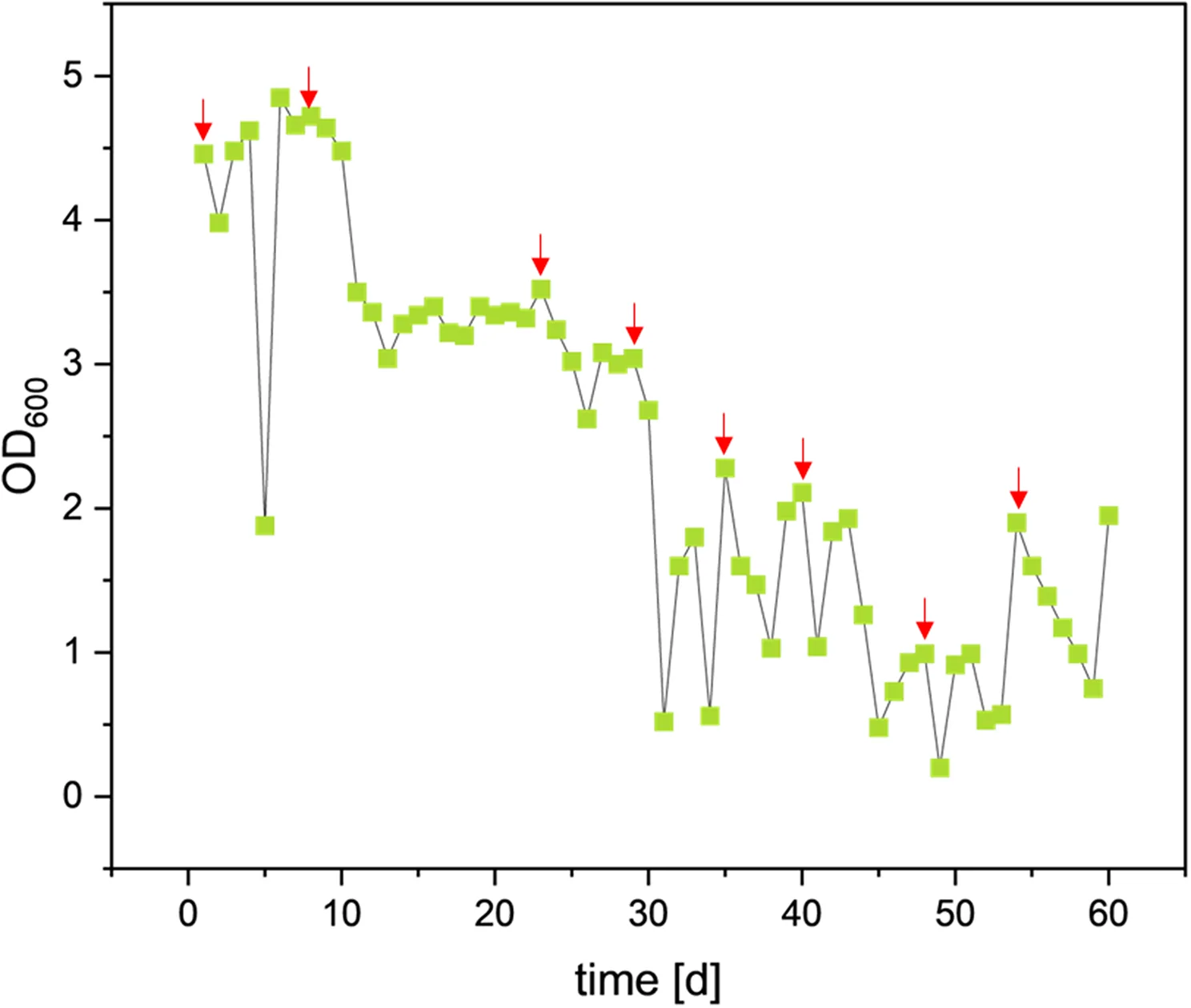
Max. OD_600_-values of the *C. necator* H16 adNaCl strain during ALE. *C. necator* H16 was adapted to NaCl (green squares) over a period of 61 days. Each day fresh medium with periodically rising salt concentrations (indicated by red arrows from left to right: 100, 200, 250, 300, 350, 400, 450 and 500 mM) was inoculated to an OD_600_ of 0.1. The OD_600_ after 24 h of incubation was measured daily and displayed here showing the overall decrease of OD_600_. The cultures were incubated at 30 °C and 180 rpm in baffled Erlenmeyer flasks. The shown data is representative for all adaptations

To generate *C. necator* variants adapted to osmotic stress, adaptive laboratory evolution (ALE) was performed through daily transfer of cells into fresh medium containing gradually increasing concentrations of NaCl or Na_2_SO_4_. Initially, every 5–7 days, 100 mM steps were used, and after 23 days, increments of 50 mM, until stable growth was achieved in minimal medium supplemented with 500 mM of NaCl or 400 mM Na_2_SO_4_. The strains were adapted in the absence of the plasmid pSEVA228-setA. This was intentional, as the transformation and activation of the transporter would enable the secretion of trehalose, whereas intracellular trehalose accumulation contributes to osmoprotection. Maintaining trehalose intracellularly during ALE therefore allowed the evolutionary selection to act on the cellular response to osmotic stress, that would later be the trigger during production. Single colonies of the adapted cultures were transformed after ALE. During ALE, the maximum daily measured OD_600_ values of the strains decreased after each increase in salt concentration but recovered during continued cultivation within 5–7 days. Once growth recovered, the salt concentration was increased again. At the beginning of the ALE process, strains reached OD_600_, _max_ values of approximately 4–5 whereas at the end of the process it reduced to around 2.3 (Fig. 2). For the ALE process of C. necator H16 to Na_2_SO_4_, a similar development of daily OD_600, max_ can be observed (Fig. S1).

**Fig. 3:**
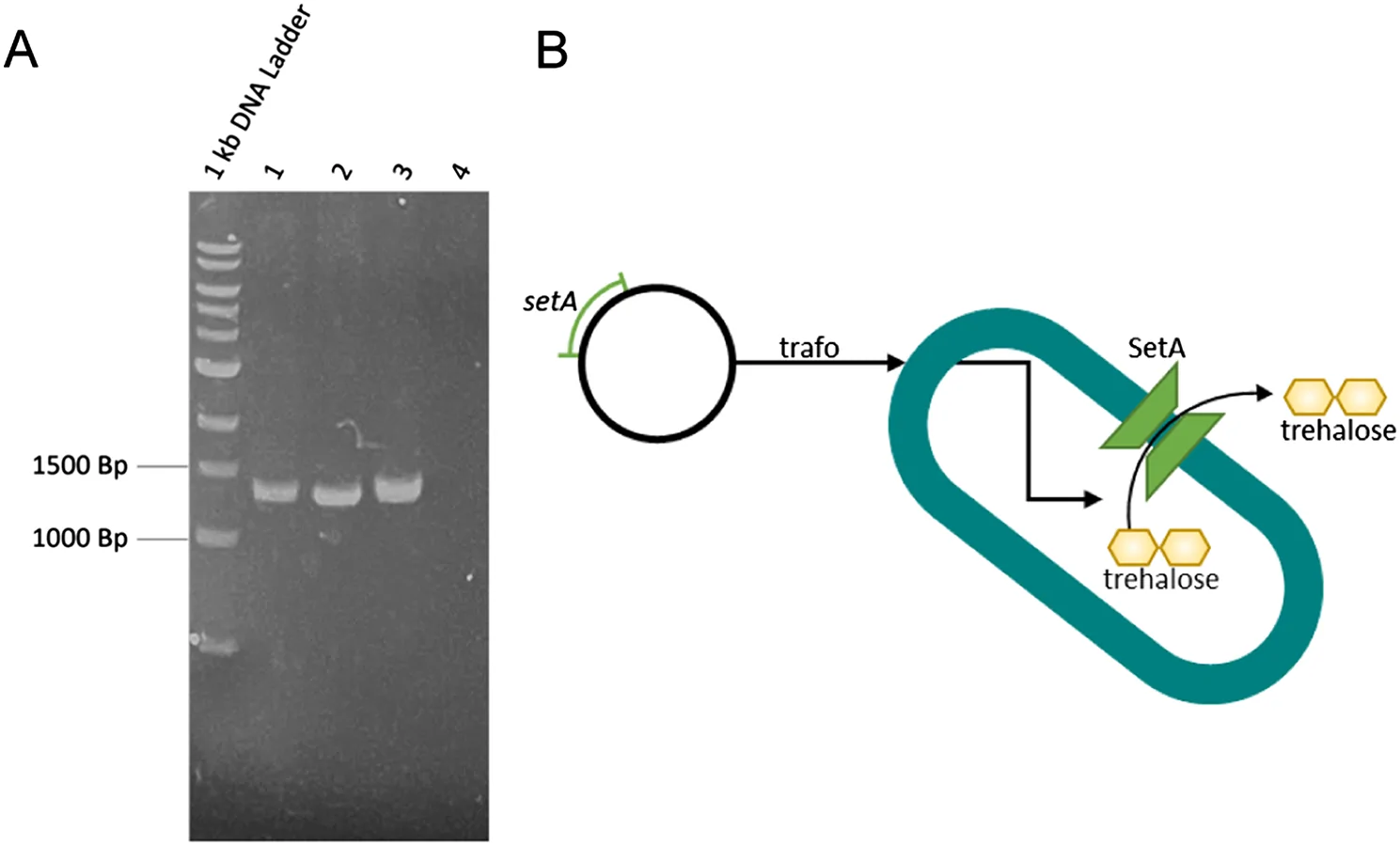
Colony-PCR of all strains transformed with pSEVA228-setA and scheme of transformation. **A**: PCR products of a colony PCR with all transformed strains were analysed via gel electrophoresis at 150 V (25 min). Samples from cultures of *C. necator* H16 SetA (1), *C. necator* H16 adNaCl SetA (2), *C. necator* H16 adNa_2_SO_4_ SetA (3), *C. necator* H16 (4) were analysed with a 1 kb DNA ladder as standard. **B**: Scheme of the transformation of the plasmid pSEVA228-setA carrying the gene of interest *setA*, which codes for the sugar efflux transporter A of *E. coli*, that’s built into the membrane and enables *C. necator* to export trehalose to the medium

Following successful ALE, isolated colonies from all adapted and parental *C. necator* H16 strains were transformed with the plasmid pSEVA228-setA, purified from *C. necator* H16 pSEVA228-setA. Both the parental strain *C. necator* H16 and adapted variants were transformed with pSEVA228-setA to enable trehalose secretion (Table 1). Colony PCR was performed to confirm the successful transformation of the strains (Fig. 3). The expected PCR product size was observed in all transformed strains. Four cultures without a stressor (LB cyro-culture, LB plate, LB liquid medium, minimal medium) were cultivated before inoculation of experimental main cultures, indicating genetic stability of the adapted strains.

To further analyse the adapted strains, the variants that were transformed with pSEVA228-setA were sequenced via Illumina sequencing. In Tables S1 and S2, the resulting mutations are listed. Genome sequencing of the selected adapted isolates identified multiple point mutations affecting different cellular functions that can be related to adaptation in high osmotic environments. In the Na_2_SO_4_-adapted isolate, mutations were identified in genes associated with regulation, transport, membrane-associated functions, energy metabolism and the respiratory chain. The regulators LysR (regulating metabolism, stress response, and transporters), as well as IclR (regulating carbon metabolism and general stress response), showed mutations [64]. Also, two ABC transporter proteins (ATP-binding and substrate-binding) which are known for solute transport across the membrane as well as the lactate permease LctP family transporter show mutations [65–67]. A third group of mutated genes playing a possible role in osmotic stress adaptation are components for membrane structure and stability, like BamD as well as HpnD. Lastly, few components of the respiratory chain and energy metabolism show mutations as well [68]. The NaCl-adapted isolate also contained mutations affecting regulatory proteins, membrane-associated functions and central metabolism. The frameshift mutation occurring in LysR points to a change in function of the regulator playing a huge role in metabolic stress response and for transporters [64]. Further mutations appeared in genes connected to peptidoglycan biosynthesis which strongly affect the thickness, stability and permeability of the cell wall during osmotic stress. Changes in isoprenoids, membrane lipids and hopanoids alter membrane fluidity as well as electron transport [65]. Other possible indirectly relevant mutations occurred in genes of the carbon metabolism [69, 70]. These adaptations illustrate the potential of ALE as an alternative to classical engineering, when the desired phenotype may depend on multiple cellular processes. The distribution of mutations across several functional categories is consistent with the intended whole-cell nature of the ALE approach. In particular, changes affecting transporters and membrane-associated functions are plausible candidates for altered osmotic stress response, whereas mutations affecting energy and central metabolism may influence the balance between growth and production. The sequencing results could be interpreted as candidate genetic changes associated with the evolved isolates rather than as validated mechanism connected to enhanced trehalose production. Transcriptomic, proteomic, intracellular metabolite or reverse engineering studies would be required to identify the molecular changes responsible for the observed phenotype.

The purpose of subsequent experiments was not reproduction of evolutionary selection conditions during production. Instead, the adapted isolates were evaluated under moderate osmotic conditions to determine whether the evolved cellular phenotype could be translated into improved extracellular trehalose production after SetA expression.

### Enhanced trehalose production

To verify that SetA enabled extracellular trehalose production, parental and adapted *C. necator* strains carrying pSEVA228-setA were cultivated without (Fig. S2) and with (Fig. S3) SetA induction in minimal medium supplemented with 0–500 mM Na_2_SO_4_. In the presence of the transporter, trehalose was detected in the cultivation medium, whereas the untransformed parental strain showed only low extracellular trehalose concentrations. These results confirm that the production phenotype measured in subsequent experiments represents extracellular trehalose enabled by heterologous SetA expression. This is best visible at the experiments with 200 and 300 mM sodium sulphate. The NaCl-adapted variant (red) shows slightly earlier maximal growth at 200 mM sodium sulphate (Fig. S2C) as well as higher and faster growth at 300 mM sodium sulphate (Fig. S2D). The effect of the expression of SetA can be seen in figure S3, showing how growth of the different strains proceeds when the cells have the possibility to secrete trehalose. Within these growth curves there are no significant differences between the tested strains. It appears as if without the possibility to use trehalose intracellular as compatible solute, the adaptation shows no effect on growth of adapted variants under the specifically used conditions compared to the parental strain. In figure S4 the growth, trehalose and fructose concentrations were measured in minimal medium with 4 g/L fructose and 150 mM sodium sulphate as osmotic trigger of adapted strains and the parental strain all transformed with pSEVA228-setA as well as of the untransformed parental strain *C. necator* H16. Cultures were inoculated at an OD_600_ of 0.1 and grown for 162 h at 30 °C and 180 rpm. It is clearly visible that, the plasmid pSEVA228-setA and the induction of SetA enabled all transformed strains to secrete trehalose at concentrations between 0.25 and 0.38 g/L, whereas the trehalose concentration of the untransformed strain stayed beneath 0.1 g/L.

Afterwards growth and trehalose production of all transformed H16-strains were analysed in minimal medium with 4 g/L fructose as carbon and energy source, supplemented with 150 mM NaCl (Fig. S5 A, D, G), Na_2_SO_4_ (Fig. S5 B, E, H) and MgSO_4_ (Fig. S5 C, F, I) as osmotic trigger to analyse the most suitable trigger for trehalose production. Cultures were inoculated to an OD_600_ of 2 and cultivated in 30 °C and 180 rpm. The OD_600_ was similar in all strains over the course of time but with a slight improvement in the presence of MgSO_4_. Trehalose concentration was consistently highest in medium supplemented with Na_2_SO_4_, where all strains reached concentrations of 0.5–0.8 g/L trehalose. In contrast, in medium supplemented with NaCl or MgSO_4_ concentrations of 0.1–0.4 g/L of trehalose were reached.

The three salts NaCl, Na_2_SO_4_ and MgSO_4_ were compared as osmotic triggers at 150 mM. Under the tested conditions, Na_2_SO_4_ resulted in the highest extracellular trehalose concentrations across the strains, whereas NaCl and MgSO_4_ resulted in lower concentrations. Based on this screening results, Na_2_SO_4_ was selected for subsequent production experiments. Because the salts were compared at equal molar rather than equal osmolar concentrations, the experiment does not constitute a comparison at equal osmotic pressure. In particular NaCl and Na_2_SO_4_ differ in their dissociation behaviour and ion composition.

Osmolarity is defined as the number of solutes per litre. NaCl and MgSO_4_ dissolve in two ions meaning two solutes (Na^+^ and Cl^−^, Mg^2+^ and SO_4_^2−^) but Na_2_SO_4_ dissolves into three solutes (2Na^+^ and SO_4_^2−^). Accordingly, the same concentration of Na_2_SO_4_ causes a higher osmolarity and therefore, put a higher osmotic burden on the cells [71]. Besides the higher osmolarity of Na_2_SO_4_ also the different anions could possibly have effects on the cellular membrane, protein and cytoplasm interactions impacting trehalose production. Cl^−^ is able to transfer the membrane and has therefore possible toxic effects like cytoplasm acidification or ROS-formation [72]. The SO_4_^2−^ form bigger hydration radii and therefore has a sole osmotic effect on the cell, no toxic effect [73]. The NaCl-adapted variant therefore adapted not just to osmotic pressure but also to stress response towards toxins.

Therefore, the present data supports the selection of Na_2_SO_4_ as the most effective trigger among the tested conditions, but does not allow the salt specific effect to be attributed exclusively to differences in osmolarity. Direct measurements of osmolarity and experiments at matched osmolarities would be required to resolve this distinction. Consequently, following experiments were conducted with Na_2_SO_4_ as osmotic trigger for the production of trehalose.

**Fig. 4:**
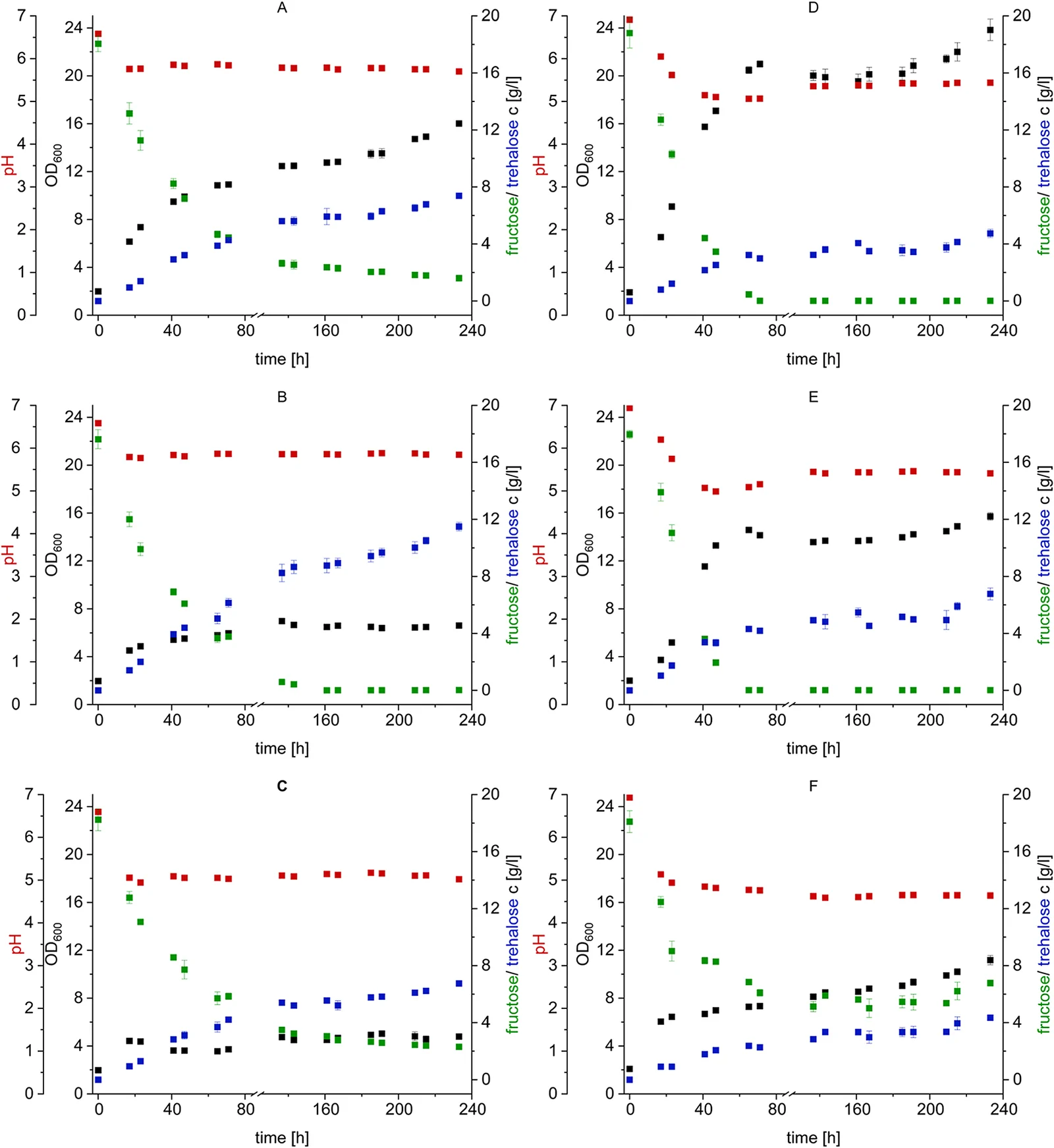
Comparison of different media for trehalose production**.** As osmotic trigger for the production of trehalose in *C. necator* H16 SetA (**A, D**) and the adapted variants *C. necator* H16 adNaCl SetA (**B, E**) and *C. necator* H16 adNa_2_SO_4_ SetA (C, F) 150 mM of Na_2_SO_4_ were used. All strains were tested in minimal medium according to Sydow (**A – C**) and the FN minimal medium (**D – F**). In both cases 20 g/L fructose as carbon and energy source were used. Cultures were incubated at 30 °C and 180 rpm for 240 h (*n* = 3)

For further screening two minimal media were compared, FN-medium commonly used for high concentrations of biomass as well as minimal medium according to Sydow et al. 2017 (MM acc. Sydow), which is a minimal medium optimized for the application in electro biotechnological applications as it combined needs of bacteria with electrochemical ones. The concentration of fructose as carbon source was enhanced from 4 g/L as in the salt screening experiments to 20 g/L in this experiment for the comparison of two media. In MM acc. Sydow Medium (Fig. 4A - C) the H16 adNaCl SetA strain (4B) produced the highest amount of trehalose up to 12 g/L whereas the parental strain (4A) as well as the H16 adNa_2_SO_4_ SetA (4 C) strain produced around 7–8 g/L trehalose. In FN-medium (Fig. 4D – F) H16 adNaCl SetA (4E) performed best with around 7 g/L trehalose, the H16 SetA (4D) as well as the H16 adNa_2_SO_4_ SetA (4 F) strain produced around 4 g/L trehalose. Analysis of media composition revealed possibilities for differences of up to 5 g/L trehalose in the best producing strain H16 adNaCl SetA. To compare growth of all strains in both media on a deeper level all yields and process metrics were calculated according to Eqs. 1–3. They are shown for all cultivations in Table 2.

**Table 2:** Process metrics of the comparison of minimal medium according to Sydow and FN minimal medium with *C. necator* H16 SetA and adapted variants (*n* = 3)

Medium	Strain	∆BTM [g/L]	∆C_fru_ [g/L]	∆C_tre_ [g/L]	Y_X/S_ [g/g]	Y_*P*⁄X_ [g/g]	Y_*P*⁄S_ [g/g]
Minimal medium acc. to Sydow	*C. necator* H16 SetA	5.09 ± 0.09	18.40 ± 0.07	7.37 ± 0.19	0.277 ± 0.006	1.45 ± 0.05	0.401 ± 0.011
	*C. necator* H16 adNaCl SetA	1.68 ± 0.04	20.00 ± 0.01	11.49 ± 0.29	0.084 ± 0.002	6.83 ± 0.23	0.575 ± 0.015
	*C. necator* H16 adNa_2_SO_4_ SetA	1.02 ± 0.074	17.69 ± 0.05	6.75 ± 0.06	0.058 ± 0.004	6.60 ± 0.48	0.382 ± 0.004
FN minimal medium	*C. necator* H16 SetA	7.96 ± 0.32	20.00 ± 0.00	4.75 ± 0.29	0.398 ± 0.016	0.59 ± 0.04	0.237 ± 0.015
	*C. necator* H16 adNaCl SetA	4.98 ± 0.12	20.00 ± 0.00	6.77 ± 0.42	0.249 ± 0.006	1.36 ± 0.09	0.339 ± 0.021
	*C. necator* H16 adNa_2_SO_4_ SetA	3.30 ± 0.14	13.22 ± 0.15	4.34 ± 0.18	0.250 ± 0.012	1.32 ± 0.08	0.329 ± 0.015

**Fig. 5:**
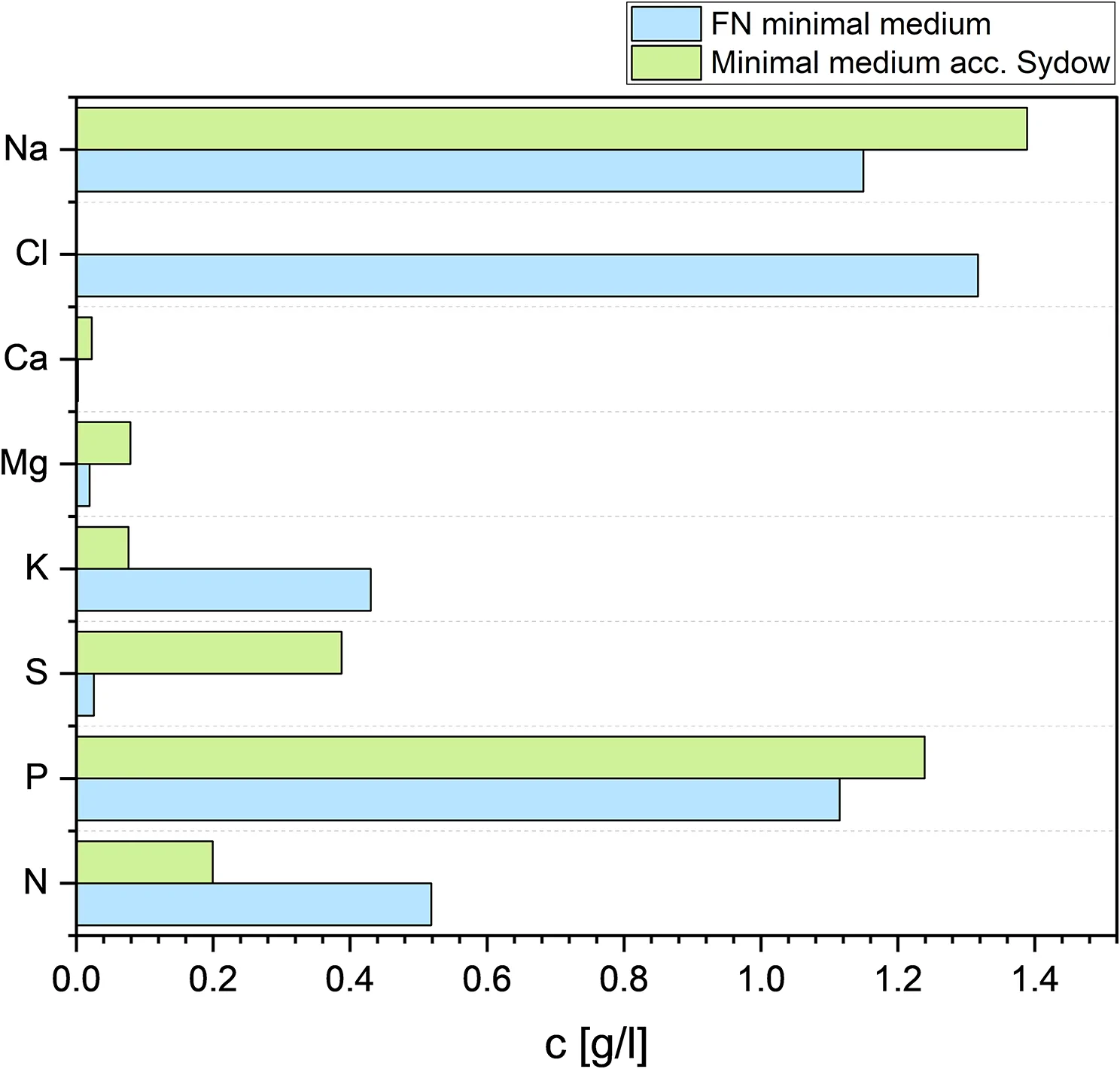
Elementary balance of both minimal media used for trehalose production with *C. necator* H16 SetA and adapted variants. Concentrations of the most important elements of both minimal media calculated according to their composition. FN-medium is displayed in blue, minimal medium according to Sydow in green bars

**Fig. 6:**
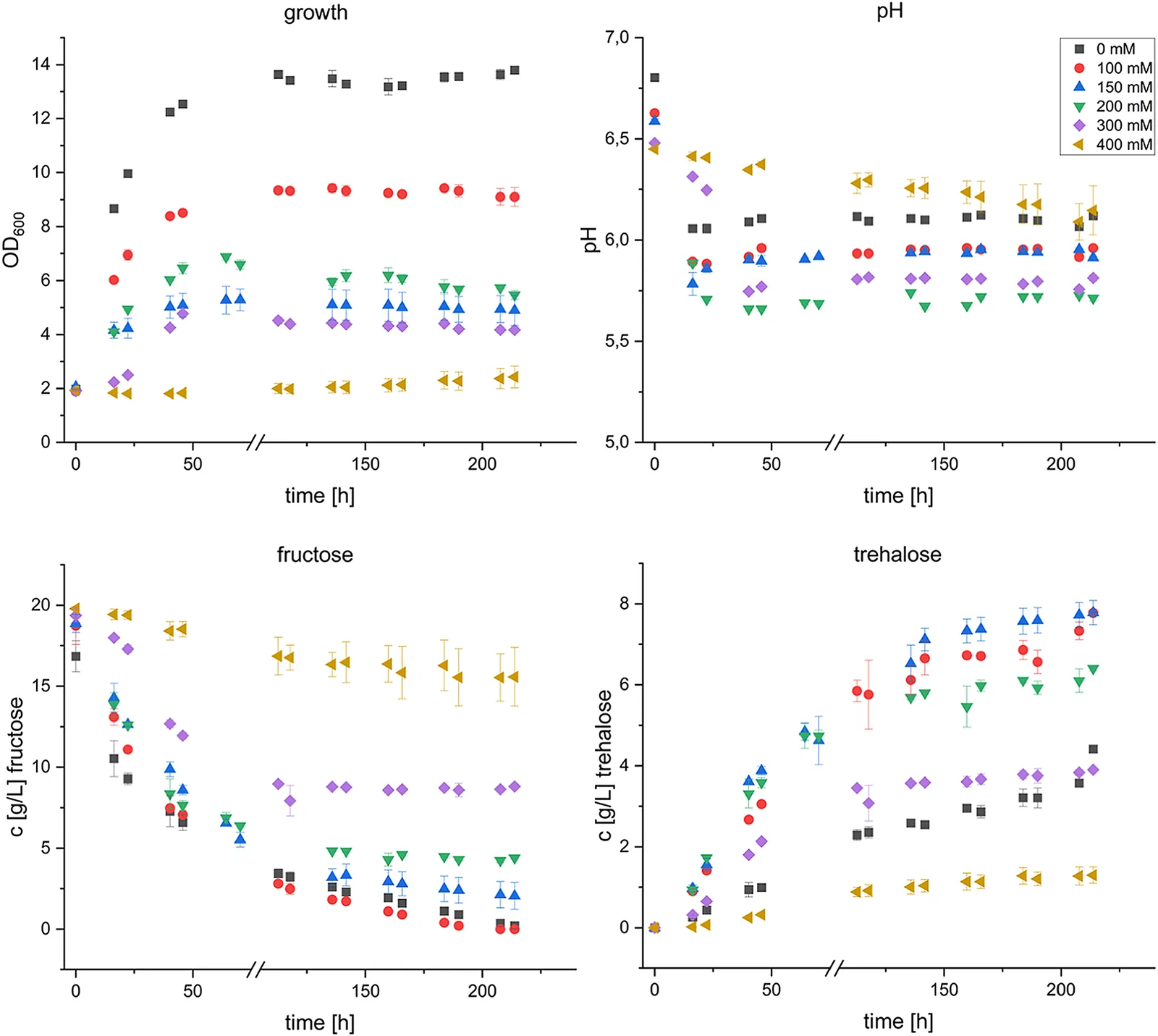
Screening of the ideal Na_2_SO_4_ concentration for trehalose production with *C. necator* H16 adNaCl SetA. *C. necator* H16 adNaCl SetA was cultivated in minimal medium with 20 g/L of fructose as carbon and energy source at 30 °C and 180 rpm. Salt resistance was measured in growth via OD_600_, pH and the consumed fructose and formed trehalose which was measured via HPLC. Concentrations of Na_2_SO_4_ of 0 (black squares), 100 (red circles), 200 (green downward triangles), 300 (purple diamonds) and 400 mM (gold leftward triangles) were tested along with the 150 mM (blue triangles) that were used initially (*n* = 3)

While comparing both minimal media (Fig. 5) it is clear that in minimal medium according to Sydow (green bars), the strains performed better concerning the production of trehalose. The FN-medium (blue bars) which is used typically as production medium for biomass performed not as good concerning the trehalose production. Although, in FN-medium strains reached at least double the OD_600_ compared to the strains grown in MM acc. Sydow. Comparing the elementary balance in figure. 6 it shows that sodium (MM acc. Sydow: 1.39 g/L, FN: 1.15 g/L) and phosphour (MM acc. Sydow: 1.24 g/L, FN: 1.12 g/L) are present in high concentrations in both media, on the other hand, the medium for electrobiotechnological experiments is free of chloride as in such experiments under certain conditions the risk of chlorine gas formation can be high. In the FN-medium chloride (1.32 g/L), potassium (0.43 g/L) and nitrogen (0.52 g/L) are enhanced compared to the MM acc. Sydow (0 g/L, 0.08 g/L, 0.2 g/L). In MM acc. Sydow calcium (0.02 g/L), magnesium (0.08 g/L) and sulphure (0.39 g/L) are present in low concentrations but higher compared to FN-medium (0 g/L, 0.02 g/L, 0.03 g/L). The low nitrogen in MM acc. Sydow could be a reason for an early nitrogen shortage in the long cultivation duration. Nitrogen starvation triggers the formation of polyhydroxybutyrate (PHB) in *C. necator* [26] and possibly also triggers the production of other storage substances like trehalose [74]. Another possibility is the ratio of sodium to potassium. In MM acc. Sydow the ratio is about 18.2, in FN-medium it’s just 2.67. In cell culture as well as other microorganisms it is known that this ratio contributes to the cell viability and the osmoadaptation as part of the “salt-in” strategy [75, 76]. The two minimal media were compared using 20 g/L fructose and 150 mM Na_s_SO_4_ as osmotic trigger. The minimal medium according to Sydwo et al. supported higher extracellular trehalose production than FN medium for all tested strains. In the Sydow medium, the NaCl adapted Set A strain reached approximately 12.g/L trehalsoe whereas the parental SetA and the Na_2_SO_4_-adapted SetA strains reeached approcimately 7–8 g/L. In FN medium, trehalose production was lower for all strains.

The higher trehalose production in the Sydow medium may reflect differneces in nutrient and ion composition. However, the present experiments were not designed to idenitfy the individual medium components responsible for this difference. The Sydow medium was therefore selected for subsequent production optimization based on its higher observed trehalose production.

The next objective was to identify a production concentration of Na_2_SO_4_ that provided sufficient osmotic induction while maintaining sufficient cellular growth and substrate conversion. H16 adNaCl SetA was therefore cultivated in minimal medium containing 20 g/L fructose as carbon and energy source with Na_2_SO_4_ (0–400 mM). Growth and trehalose production were measured during cultivation.

Without additional Na_2_SO_4_ (Fig. 6. black squares) the culture reached a maximum OD_600_ of 14 and produced approximately 4 g/L trehalose. The substrate fructose was fully consumed just at the end of the fermentation. Addition of 100 mM of Na_2_SO_4_ (Fig. 6. red circles), reduced the OD_600, max_ to 9, but increased trehalose production to concentration of 8 g/L, fructose was also fully consumed by the end of the cultivation. At 150 mM of Na_2_SO_4_ (Fig. 6. blue triangle), a similar concentration of trehalose was obtained, while growth was further reduced and fructose wasn’t fully consumed leaving about 2 g/L at the end of the cultivation. At 200 (Fig. 6. green triangles), 300 mM Na_2_SO_4_ (Fig. 6. purple diamonds) maximum reached OD_600_ and the fructose consumption decrease further, trehalose production declined to approximately 7 and 4 g/L, respectively. At 400 mM Na_2_SO_4_ (Fig. 6. gold triangles) no growth could be observed. Fructose consumption was limited to 4 g/L.

Among the concentrations tested, an osmotic trigger of 150 mM Na_2_SO_4_ was therefore selected as favoured production condition, as it provided a high trehalose concentration while maintaining measurable growth. The results also demonstrate that increasing osmotic stress does not continuously increase trehalose production. At higher salt concentrations, the inhibitory effect on growth and substrate conversion outweighed the potential benefit of a stronger osmotic stimulus. The yields and growth rates of all cultivations are shown in table S3 and calculated according to Eqs. 1–3. The best condition for trehalose production is 150mM Na_2_SO_4_ with around 8 g/L of trehalose, but the growth of cultures with 100 mM and 200 mM is higher and also substrate consumption of the 100 mM culture is higher compared to the culture supplemented with 150 mM Na_2_SO_4_. Consequently, the yields of product per biomass and per substrate are highest under these conditions.

In this study we demonstrated the beneficial effects of adaptive laboratory evolution to sodium salt stress on the enhanced production of trehalose. The adaptation process was carried out with high final concentrations of NaCl (500 mM) or Na_2_SO_4_ (400 mM). However, following the approach of Löwe et al., lower concentrations proved to be more suitable for production experiments. The theoretical max. yield of trehalose from fructose is 1 mol trehalose per 2 mol fructose without accounting for biomass formation or energy metabolism. Accordingly, 20 g/L fructose (111 mM) theoretically yield 19 g/L trehalose (55.5 mM). In this study a maximum trehalose concentration of 11.5 g/L (33.57 mM) was achieved, corresponding to 60.5% of the theoretical maximum. In comparison, the non-adapted strain which produced only 7.37 g/L trehalose from 18.4 g/L fructose, corresponding to 42.1% of the theoretically max. yield. Because the comparison was performed using adapted isolates rather than multiple independently evolved lineages, these data demonstrate the improved production phenotype of an isolate but do not establish that the phenotype is reproducible across independent ALE lineages. Therefore, under the tested production conditions the NaCl-adapted isolate achieved an 18.4% points higher fraction of the theoretical max. trehalose yield than the parental SetA strain (60.5% vs. 42.1%). As another comparative variable the volumetric productivity of both strains was calculated with Eq. 4. The NaCl-adapted isolate achieved during the media comparison 11.5 g/L over a duration of 240 h resulting in a vol. productivity of 0.048 g/L/h, compared to 0.031 g/L/h. The adapted strain achieved therefore a 56% higher volumetric productivity, nevertheless this also highlights the extremely long duration of the cultivation. The differences represent the production phenotype observed for the selected adapted isolate under specific conditions and should not be interpreted as a general measure of the reproducibility of ALE. Additionally, to the increased percentage of the theoretically max. yield that was reached by the adapted strain compared to the parental strain, the best performance in carbon yield was also reached in the pre-experiment (Table 2). By adding up the amount of biomass and product and subtracting it of the consumed substrate a residual amount of substrate that was not transformed into product or biomass is uncovered. This part of the substrate is not used for the production of the dedicated product, but rather for unaccounted sinks. The carbon yield of both, the parental strain (H16 SetA) as well as the NaCl adapted variant (H16 adNaCl SetA) lies between 65 and 68%. Therefore around 32–35% of the substrate are used not for trehalose production but the maintenance metabolism or unknown sinks like organic acid production instead. To uncover such potential sinks could also help enhancing the possible output by the adapted host cells of the desired trehalose. Nevertheless, it also shows, that the carbon yield of the adapted and the parental strain don’t differ rather much although the amount of biomass and trehalose produced by the strains do, showing that a shift of substrate usage happened. The H16 SetA produced more biomass and less trehalose whereas H16 adNaCl SetA produced more trehalose and less biomass. The observed shift from biomass formation toward extracellular trehalose production may reflect changes in the balance between growth, stress adaptation and carbon allocation. The selected NaCl-adapted isolate produced substantially more trehalose while forming less biomass than the parental strain, whereas the overall carbon balance remained within a similar range. This observation is consistent with a redistribution of substrate between biomass formation and product formation, but underlying metabolic mechanisms cannot be resolved with the present data. The genome sequencing revealed mutations in several regulators, transporters, proteins associated with membrane stability and metabolic processes. These changes provide plausible candidate mechanisms for altered osmoadaptation or carbon allocation, but no direct causal relationship with trehalose biosynthesis was established. In particular, intracellular trehalose and other compatible solutes were not quantified. Therefore, the present study demonstrates an altered production phenotype rather than a defined molecular mechanism.

## Conclusion

In the case of this study without being able to identify a global goal for rational engineering beforehand the enhancement of trehalose production was achieved via ALE. Hereby, it was taken advantage of the methods possibility to alter an entire organism and adapt it to certain conditions – something impossible to achieve with rational engineering of single genes in about the same time. In this study, ALE was used as a whole-cell engineering strategy to adapt *C. necator* H16 to increasing osmotic stress. Two independent ALE lineages were initiated for each salt condition, and adaptation was performed in the absence of the heterologous trehalose exporter SetA. This design selected for cellular adaptation to increasing salt concentrations rather than directly for extracellular trehalose production, that would counteract the natural cause for the production of a *de novo* synthesised solute. Alternatively, in future research ALE in the presence of SetA would be interesting, to confirm whether trehalose is produced or not, when being secreted at the same time. Following adaptation, individual colonies were isolated and equipped with SetA to enable trehalose secretion. The evolutionary and production conditions served different purposes. High salt concentrations were used during ALE as selective pressure, whereas moderate salt concentrations were subsequently evaluated as production trigger to balance the stress response induction with growth and substrate conversion. Among the conditions tested, 150 mM Na_2_SO_4_ provided a favourable production condition for the selected *C. necator* H16 adNaCl SetA isolate. Under the selected conditions, *C. necator* H16 adNaCl SetA produced 11.49 ± 0.29 g/L trehalose from 20.00 ± 0.01 g/L fructose, corresponding to 60.5% of the theoretical max. yield. *C. necator* H16 SetA produced 7.37 ± 0.19 g/L trehalose and reached 42.1% of the theoretical maximum. Thus, the selected adapted isolate showed improved extracellular trehalose production compared with the parental strain under the here tested conditions.

Trehalose is a commonly occurring compatible solute used in osmoadaptation, which is a widespread process across many organisms and involves multiple regulatory steps. Trehalose is generally produced in later stages of the cascade-like production of solutes [14], as its *de novo* synthesis requires substantial amounts of energy and carbon, thereby reducing availability for growth and maintenance metabolism. Consequently, following the adaptation to sodium salts, several stages of this cascade may contribute to the enhanced trehalose production observed in this study that could only be identified by future transcriptomics or proteomics. Nevertheless, genome sequencing identified mutations affecting several cellular functions, including transport, membrane-associated processes, regulation and metabolism. These changes support the interpretation of a multifunctional whole-cell adaptation, but their individual contribution to the observed phenotype remains unsolved at the moment. Intracellular trehalose and other compatible solutes were not quantified, and identified mutations were not experimentally validated.

To further optimize the trehalose production with *C. necator* host cells with the minimal medium according to Sydow et al. (2017) medium optimization is a promising option. Components like nitrogen can have big impacts on production processes as a nitrogen starvation can be a requirement for e.g., PHB production [77]. Another target for optimization might be the Na/K-ratio. In yeast or cell culture this ratio was already shown to have an impact on cell viability and osmo-tolerance as part of the “salt in”-strategy [75, 76]. Another possible and worth mentioning trigger for osmotic stress next to the much-discussed salts is the carbon source itself. High concentrations of sugar are not often talked about in the context of osmotic pressure but are proven to have an effect [78]. Overall, the results demonstrate the potential of salt-stress ALE as a whole cell engineering strategy for generating *C. necator* isolates with improved extracellular trehalose production. This study does not establish the reproducibility of this phenotype across independent ALE lineages or a direct molecular mechanism linking the identified mutations to trehalose production. Further studies using independently evolved and characterised lineages, intracellular metabolite measurements and molecular validation would be required to determine the generality and mechanism of the observed phenotype. The combination of the approaches of Löwe et al., showing trehalose production during autotrophic cultivation, and the herein shown ALE to sodium salts also remains to be investigated.

## Supplementary Information

Below is the link to the electronic supplementary material.

Supplementary Material 1

## Data Availability

The data that support the findings of this study are available from the corresponding author upon reasonable request. Sequencing data is available at ENA (Accession: PRJEB127622).
