# Supplementary material for: Adaptive laboratory evolution of Cupriavidus necator to sodium salts as a whole cell engineering strategy for enhanced trehalose production
Source: Microb Cell Fact. 2026 Nov 7;25:238. doi: 10.1186/s12934-026-03167-8 (PMC13757053; doi:10.1186/s12934-026-03167-8)
Supplement: Supplementary file 1 [file 12934_2026_3167_MOESM1_ESM.docx]

**Supplementary Information**

**Figure S1.: Max. OD_600_-values of the C. necator H16 adNa_2_SO_4_ strain during ALE.** C. necator H16 was adapted to Na_2_SO_4_ (blue circles) over a period of 58 days. Each day fresh medium with periodically rising salt concentrations (indicated by red arrows from left to right: 100, 200, 250, 300, 350, and 400 mM) was inoculated to an OD_600_ of 0.1. The OD_600_ after 24 h of incubation was measured daily and displayed here showing the overall decrease of OD_600_. The cultures were incubated at 30 °C and 180 rpm in baffled Erlenmeyer flasks.

 **Figure S2.: Growth comparison of C. necator H16 SetA and adapted variants without induction in different Na_2_SO_4_ concentrations.** As osmotic trigger for the production of trehalose in C. necator H16 SetA (black) and the adapted variants C. necator H16 adNaCl SetA (red) and C. necator H16 adNa_2_SO_4_ SetA (blue) were grown in minimal medium with 4 g/L fructose as carbon and energy source supplemented with 0, 100, 200, 300, 400, 500 mM (A-F) of Na_2_SO_4_. The cultures were incubated at 30 °C and 500 rpm for 80 hours.

 **Figure S3.: Growth comparison of C. necator H16 SetA and adapted variants with induction in different Na_2_SO_4_ concentrations.** As osmotic trigger for the production of trehalose in C. necator H16 SetA (black) and the adapted variants C. necator H16 adNaCl SetA (red) and C. necator H16 adNa_2_SO_4_ SetA (blue) were grown in minimal medium with 4 g/L fructose as carbon and energy source supplemented with 0, 100, 200, 300, 400, 500 mM (A-F) of Na_2_SO_4_. The translation of the sugar efflux transporter A was induced with 0.1 mM 3-methylbenzoate (3-MB) The cultures were incubated at 30 °C and 500 rpm for 80 hours.

**Figure S4.: Comparison of C. necator H16 with transformed and/or adapted variants in minimal medium with 150 mM Na_2_SO_4_.** As osmotic trigger for the production of trehalose in C. necator H16 (green) as well as with pSEVA228-setA transformed (black) and NaCl- (red) or Na_2_SO_4_-adapted variants (blue) were grown in minimal medium with 4 g/L fructose as carbon and energy source supplemented with 150 mM of Na_2_SO_4_., 300 µM kanamycin and 0.1 3-MB. The cultures were incubated at 30 °C and 180 rpm for 162 hours. Growth as measured per OD_600_ is shown left (A), fructose consumption and trehalose formations are shown on the right (B).

**Figure S5.: Comparison of different salts as osmotic trigger for trehalose production.** As osmotic trigger for the production of trehalose in C. necator H16 SetA (A – C) and the adapted variants C. necator H16 adNaCl SetA (D – F) and C. necator H16 adNa_2_SO_4_ SetA (G – I) 150 mM of different salts were tested. NaCl (A, D, G), Na_2_SO_4_ (B, E, H) and MgSO_4_ (C, F, I) were added to the FN minimal medium with 4 g/L of fructose as carbon and energy source. The cultures were incubated at 30 °C and 180 rpm for 140 hours.

| **Table S1 Point mutations according to sequencing results of *C. necator* H16 adNa_2_SO_4_ 5** | | |
| --- | --- | --- |
| (TGC)_4→3_ | coding (1361‑1363/1761 nt) | phosphoenolpyruvate‑‑protein phosphotransferase |
| G→A | intergenic (‑98/‑191) | recombinase family protein/hemerythrin domain‑containing protein |
| C→T | W224* (TGG→TGA) | hypothetical protein |
| T→C | intergenic (+238/‑365) | MaoC family dehydratase/ubiquinol oxidase subunit II |
| G→A | I293I (ATC→ATT) | helix‑turn‑helix transcriptional regulator |
| G→A | R91H (CGC→CAC) | ABC transporter ATP‑binding protein |
| G→A | M173I (ATG→ATA) | alpha/beta fold hydrolase |
| G→A | A384T (GCC→ACC) | ATP‑dependent DNA helicase |
| G→A | A64V (GCC→GTC) | outer membrane protein assembly factor BamD |
| G→A | W107* (TGG→TAG) | class I poly(R)‑hydroxyalkanoic acid synthase |
| G→A | L74L (CTC→CTT) | type II toxin‑antitoxin system RelE/ParE family toxin |
| G→A | S10F (TCC→TTC) | type II toxin‑antitoxin system RelE/ParE family toxin |
| G→A | intergenic (+125/‑42) | hypothetical protein/cystathionine beta‑lyase |
| C→T | intergenic (‑110/+8) | DUF2789 domain‑containing protein/PepSY domain‑containing protein |
| G→A | R276C (CGC→TGC) | presqualene diphosphate synthase HpnD |
| C→T | D234N (GAC→AAC) | YdiU family protein |
| G→A | D282N (GAT→AAT) | 3‑hydroxyacyl‑CoA dehydrogenase NAD‑binding doma in‑containing protein |
| G→A | G160D (GGT→GAT) | LysR family transcriptional regulator |
| G→C | L28F (TTG→TTC) | alpha/beta fold hydrolase |
| G→A | T459M (ACG→ATG) | cytochrome c oxidase accessory protein CcoG |
| G→A | G238D (GGC→GAC) | GNAT family N‑acetyltransferase |
| G→A | L118L (CTG→TTG) | succinate dehydrogenase, cytochrome b556 subunit |
| A→C | D53E (GAT→GAG) | 1‑deoxy‑D‑xylulose‑5‑phosphate synthase |
| G→A | A98T (GCC→ACC) | flavin reductase family protein |
| C→T | G147S (GGC→AGC) | IclR family transcriptional regulator |
| G→A | intergenic (+236/‑7) | aspartate 1‑decarboxylase/NAD(P) transhydrogenase subunit alpha |
| C→G | E179D (GAG→GAC) | glutamate‑5‑semialdehyde dehydrogenase |
| C→T | E187K (GAA→AAA) | DGQHR domain‑containing protein |
| C→T | intergenic (+95/‑115) | dephospho‑CoA kinase/cell division protein ZapD |
| (GGC)_3→2_ | coding (298‑300/1341 nt) | histidinol dehydrogenase |
| T→C | H131R (CAC→CGC) | 30S ribosomal protein S3 |
| G→A | K15K (AAG→AAA) | D‑2‑hydroxyacid dehydrogenase family protein |
| G→A | intergenic (+181/‑57) | response regulator transcription factor/response regulator transcription factor |
| C→T | A157T (GCG→ACG) | lactate permease LctP family transporter |
| G→A | G17S (GGC→AGC) | PAAR domain‑containing protein |
| A→T | N443K (AAT→AAA) | ABC transporter substrate‑binding protein |
| G→T | G197G (GGC→GGA) | tripartite tricarboxylate transporter substrate binding protein |
| T→C | T57T (ACT→ACC) | HigA family addiction module antitoxin |
| C→T | G465D (GGC→GAC) | amino acid adenylation domain‑containing protein |
| C→T | G148D (GGT→GAT) | 2‑hydroxycyclohexanecarboxyl‑CoA dehydrogenase |
| A→G | pseudogene (976/1102 nt) | IS5 family transposase |
| A→G | C286C (TGT→TGC) | form I ribulose bisphosphate carboxylase large s ubunit |

| **Table S2 Point mutations and deletions according to sequencing results of *C. necator* H16 adNaCl 5** | | |
| --- | --- | --- |
| T→G | I150L (ATC→CTC) | folate‑binding protein YgfZ |
| A→C | D53E (GAT→GAG) | 1‑deoxy‑D‑xylulose‑5‑phosphate synthase |
| Δ1 bp | coding (579/996 nt) | LysR family transcriptional regulator |
| A→G | L78P (CTG→CCG) | phospho‑N‑acetylmuramoyl‑pentapeptide‑ transferase |
| T→C | H131R (CAC→CGC) | 30S ribosomal protein S3 |
| C→T | V256M (GTG→ATG) |  |
| C→T | R700C (CGC→TGC) | acetate‑‑CoA ligase family protein |
| C→A | T252T (ACC→ACA) | bifunctional aconitate hydratase 2/2‑methylisoci trate dehydratase |
| T→C | T57T (ACT→ACC) | HigA family addiction module antitoxin |
| C→A | R2462L (CGC→CTC) | non‑ribosomal peptide synthetase |
| T→C | L172P (CTT→CCT) | hypothetical protein |
| A→G | C286C (TGT→TGC) | form I ribulose bisphosphate carboxylase large s ubunit |

| **Table S3.: Process metrics** of the comparison of different Na_2_SO_4_ concentrations in minimal medium according to Sydow with *C. necator* H16 adNaCl SetA (n = 3) | | | | | | |
| --- | --- | --- | --- | --- | --- | --- |
| **Na_2_SO_4_ concentration [mM]** | **∆BTM g/L** | ***∆C*_fru_ g/L** | ***∆C*_tre_ g/L** | **Y_X/S_ [g/g]** | **Y_P⁄X_ [g/g]** | **Y_P⁄S_ [g/g]** |
| 0 | 4.45 ± 0.28 | 19.79 ± 0 | 4.41 ± 0.02 | 0.225 ± 0.001 | 0.99 ± 0.06 | 0.223 ± 0.003 |
| 100 | 2.69 ± 0.16 | 20.00 ± 0 | 7.78 ± 0.04 | 0.134 ± 0.001 | 2.89 ± 0.17 | 0.389 ± 0.002 |
| 150 | 1.16 ± 0.36 | 17.96 ± 0.84 | 7.78 ± 0.30 | 0.065 ± 0.002 | 6.71 ± 2.11 | 0.433 ± 0.061 |
| 200 | 1.89 ± 0.14 | 15.6 ± 0.06 | 6.40 ± 0.06 | 0.41 ± 0.004 | 3.39 ± 0.25 | 0.121 ± 0.009 |
| 300 | 1.26 ± 0.18 | 11,19 ± 0,4 | 3.90 ± 0.03 | 0.113 ± 0.002 | 3.09 ± 0.45 | 0.349 ± 0.006 |
| 400 | 0.24 ± 0.45 | 5,2 ± 2,3 | 1.30 ± 0.20 | 0.055 ± 0.011 | 5.35 ± 10.29 | 0.293 ± 0.012 |
